# Does stress perfusion imaging improve the diagnostic accuracy of late gadolinium enhanced cardiac magnetic resonance for establishing the etiology of heart failure?

**DOI:** 10.1186/s12872-017-0529-y

**Published:** 2017-04-08

**Authors:** Gaurav S. Gulsin, Abishek Shetye, Jeffrey Khoo, Daniel J. Swarbrick, Eylem Levelt, Florence Y. Lai, Iain B. Squire, Jayanth R. Arnold, Gerry P. McCann

**Affiliations:** 1 Department of Cardiovascular Sciences, University of Leicester, Glenfield Hospital, Groby Road, Leicester, LE3 9QP UK; 2 The NIHR Leicester Cardiovascular Biomedical Research Unit, Glenfield Hospital, Leicester, UK

**Keywords:** Cardiovascular magnetic resonance, Heart failure, Late gadolinium enhancement, Adenosine stress perfusion, Non-ischemic cardiomyopathy

## Abstract

**Background:**

Late gadolinium enhanced cardiovascular magnetic resonance (LGE-CMR) has excellent specificity, sensitivity and diagnostic accuracy for differentiating between ischemic cardiomyopathy (ICM) and non-ischemic dilated cardiomyopathy (NICM). CMR first-pass myocardial perfusion imaging (perfusion-CMR) may also play role in distinguishing heart failure of ischemic and non-ischemic origins, although the utility of additional of stress perfusion imaging in such patients is unclear. The aim of this retrospective study was to assess whether the addition of adenosine stress perfusion imaging to LGE-CMR is of incremental value for differentiating ICM and NICM in patients with severe left ventricular systolic dysfunction (LVSD) of uncertain etiology.

**Methods:**

We retrospectively identified 100 consecutive adult patients (median age 69 years (IQR 59–73)) with severe LVSD (mean LV EF 26.6 ± 7.0%) referred for perfusion-CMR to establish the underlying etiology of heart failure. The cause of heart failure was first determined on examination of CMR cine and LGE images in isolation. Subsequent examination of complete adenosine stress perfusion-CMR studies (cine, LGE and perfusion images) was performed to identify whether this altered the initial diagnosis.

**Results:**

On LGE-CMR, 38 patients were diagnosed with ICM, 46 with NICM and 16 with dual pathology. With perfusion-CMR, there were 39 ICM, 44 NICM and 17 dual pathology diagnoses. There was excellent agreement in diagnoses between LGE-CMR and perfusion-CMR (κ 0.968, p<0.001). The addition of adenosine stress perfusion images to LGE-CMR altered the diagnosis in only two of the 100 patients.

**Conclusion:**

The addition of adenosine stress perfusion-CMR to cine and LGE-CMR provides minimal incremental diagnostic yield for determining the etiology of heart failure in patients with severe LVSD.

## Background

Identifying the etiology of heart failure has important management and prognostic implications [1]. Therapeutic strategies for ischemic cardiomyopathy (ICM) include revascularisation and/or secondary prevention measures such as antiplatelet and lipid lowering therapies. Conversely, the management of non-ischemic dilated cardiomyopathy (NICM) relies predominantly on pharmacological agents and device therapy [2]. Rarer causes of NICM (e.g. sarcoid, amyloid, HIV) require treatment of the underlying condition [3]. Importantly survival rates are poorer in those patients with heart failure of ischemic origin [4]. Clinical guidelines suggest echocardiography as the initial investigation of choice for evaluation of chronic heart failure [1, 5]. In many instances, echocardiography may reliably establish the underlying cause of heart failure. Myocardial regional wall motion abnormalities alone, however, may not distinguish heart failure of ischemic origin from NICM, since segmental wall motion abnormalities may accompany both [6]. Where echocardiography does not clearly identify the etiology of chronic heart failure, cardiac magnetic resonance imaging (CMR) is advantageous [1], owing to its capacity to detect evidence of myocardial infarction and non-ischemic fibrosis and assess ischemia and viability in a single examination [7].

**Fig. 1:**
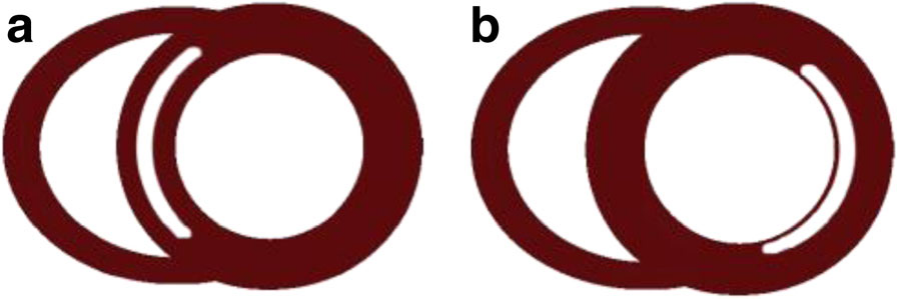
Illustration of typical patterns of LGE seen in NICM and ICM. White areas within the myocardium represent LGE. **a** Mid-wall LGE is commonly seen in NICM, whereas **b** a subendocardial distribution of LGE is typical in ICM

Late gadolinium enhanced CMR (LGE-CMR) has been shown to be non-inferior (and indeed may be superior) to coronary angiography in differentiating heart failure due to coronary artery disease (CAD) from NICM [8, 9]. Subendocardial LGE is present in CAD, whereas patients with NICM have either no LGE or mid-wall late enhancement that does not correspond to a coronary artery territory (Fig. 1) [9]. Patients with heart failure and a history of ischemic heart disease have a high prevalence of LGE visible on CMR (88% in one report) [10]. This suggests a high likelihood that LGE-CMR will yield a diagnosis of ICM by hyperenhancement patterns alone, without the need for additional perfusion imaging. Following myocardial infarction, the presence of LGE affecting ≤50% of the thickness of the myocardium predicts the likelihood of functional recovery in response to revascularisation of the affected coronary territory [11, 12]. In NICM, the presence of mid-wall fibrosis is also of prognostic value, being an independent predictor of cardiovascular and all-cause mortality [13].

CMR first-pass myocardial perfusion imaging (perfusion-CMR) has been shown in several large studies to have excellent sensitivity and specificity for detection of CAD and may thus play a role in distinguishing heart failure of ischemic and non-ischemic origins [14, 15]. However, the added value of adenosine stress perfusion-CMR in subjects with severe left ventricular systolic dysfunction (LVSD) has not been investigated previously. Given that LGE-CMR alone has excellent specificity (96%), sensitivity (100%) and diagnostic accuracy (95%) for differentiating between ICM and NICM [8], the utility of additional of stress perfusion imaging in such patients is questionable.

Adenosine stress perfusion-CMR imaging adds time (approximately 15 min added time for the perfusion-CMR protocol) and expense to the CMR protocol, due to the need for additional electrocardiograms, adenosine preparations, infusion pumps and lines, as well as physician-supervision. Undesirable symptoms and potentially serious complications may occur with adenosine infusion, albeit infrequently [16–19]. These include wheeze secondary to bronchospasm, Mobitz II 2nd or 3rd degree atrioventricular block, and angina requiring sublingual nitrates [17]. Furthermore, in patients with heart failure, there is impairment of adenosine receptor expression and signal-transduction [20], which may diminish the hemodynamic response for stress perfusion assessment, compromising diagnostic confidence.

In our regional cardiac centre, over 1000 clinical perfusion-CMR studies are performed annually. Approximately 10% of these are to identify the cause of heart failure, and despite a lack of evidence of additional benefit, stress perfusion is usually requested and performed.

The aim of this retrospective study was to assess whether the addition of adenosine stress perfusion imaging to LGE-CMR is of incremental value for differentiating ICM and NICM in patients with severe LVSD of uncertain etiology.

## Methods

### Study population

We retrospectively identified 100 consecutive adult patients with severe LVSD on echocardiography, referred for perfusion-CMR to ascertain the underlying etiology of heart failure. Patients were scanned between April 2015 and March 2016. Severe LVSD was defined as a left ventricular ejection fraction (LV EF) ≤35% and was confirmed by CMR volumetric and functional analysis prior to inclusion in the study. Participants’ past medical history, medications, electrocardiographic data, resting pulse and blood pressure were recorded at the time of the CMR.

The study was approved as a clinical audit and ethics approval was deemed unnecessary.

#### CMR image acquisition

Clinical perfusion-CMR studies were undertaken on either a 1.5 T (Siemens Avanto, Erlangen, Germany) or 3 T scanner (Siemens Skyra, Erlangen, Germany). Cardiac volumes and function were performed using standard CMR techniques as previously described by our group [21]. For stress perfusion, adenosine (140mcg/kg/min, increased up to a maximum of 210mcg/kg/min to achieve a satisfactory haemodynamic stress response) [18] was infused for 3–5 min. Patients underwent pulse, blood pressure and pulse oximetry monitoring at baseline and at 1-min intervals during adenosine infusion. Symptomatic response to adenosine was documented. During peak stress a bolus of gadolinium-based contrast (Gadoterate meglumine, Dotarem, Guerbet LLC, France) was injected (0.1 mmol/kg at 1.5 T and 0.075 mmol/kg at 3 T), followed by a 20 mL bolus of normal saline, at a rate of 5 mL/s and perfusion images were acquired using a saturation recovery gradient echo pulse sequence. Adenosine infusion was then discontinued and a complete short axis cine stack was performed before rest perfusion images were acquired following administration of a second bolus of gadolinium-based contrast agent (total dose 0.15 mmol/kg). LGE images in 3 long axis views and a complete short axis stack were acquired after a further delay of 5–10 min.

#### CMR image analysis

Scans were anonymized and sent to a separate workstation for analysis which was performed blinded to all patient details, by two specialists in CMR (JRA and JK) as recommended by expert consensus [22]. No clinical information was made available prior to or during image analysis. Definitions for etiology of severe LVSD were pre-defined as “ischemic”, “non-ischemic” or “dual-pathology” (having both ischemic and non-ischemic components and where the extent of infarction and/or ischemia did not explain the degree of LVSD). Image quality was rated as “good”, “moderate”, “poor” or “not-analyzable” for each modality. A two-stage process was employed for image analysis. The etiology of severe LVSD was determined first determined by consensus between JRA and JK after examination of cine and LGE images. Following this, the perfusion sequences were interpreted with the cine and LGE images and it was recorded whether the etiology of LVSD changed depending on the presence of reversible perfusion defects.

#### Invasive coronary angiography

Coronary angiograms were examined in those patients in the cohort who underwent angiography on clinical grounds. Images were analyzed blinded to patient details and CMR image interpretation results by an experienced cardiologist (GPM). Coronary artery disease was pre-defined as being present if a coronary artery stenosis of ≥50% the luminal diameter of the artery was observed and was noted as severe if stenosis severity was >70%.

#### Statistical analyses

Normality was assessed using Kolmogorov-Smirnov tests, histograms, and Q-Q plots. Continuous data were expressed as mean ± standard deviation, if normally distributed. Non-parametric variables were expressed as median and interquartile range. One-way analysis of variance (ANOVA) was used to compare normal data and the Independent-Samples Kruskal-Wallis test for non-normal data between groups. The Kappa statistic was used as a measure of agreement between the components of the CMR scans with and without stress perfusion. Statistical analysis was undertaken using SPSS version 20.0 software.

## Results

### Baseline characteristics

The study group consisted of 100 patients referred for clinical stress perfusion-CMR to investigate the etiology of severe LVSD. Demographic characteristics and CMR volumetric and functional data are shown in Table 1. The mean LV EF of the cohort was 26.6 ± 7.0% and there were no significant differences in LV function or volumes between patients with a diagnosis of ICM, NICM or dual pathology (Table 1).

**Table 1:** Baseline characteristics of the 100 study participants

	All patients (*n* = 100)	ICM (*n* = 39)	NICM (*n* = 44)	Dual pathology (*n* = 17)	*P*-value
Median age (years)	69 (59–73)	69 (54–84)	69 (50–88)	69 (57–81)	0.984
Gender	77% M, 33% F	77% M, 33% F	70% M, 30% F	94% M, 6% F	
SBP (mmHg)	134.7 ± 23.7	135.9 ± 24.4	132.0 ± 19.8	134.5 ± 30.7	0.715
DBP (mmHg)	79.9 ± 14.8	79.7 ± 13.2	80.3 ± 14.5	77.6 ± 20.2	0.824
Pulse rate (beats/min)	72.3 ± 14.1	69.9 ± 12.4	75.1 ± 16.3	71.9 ± 11.4	0.263
ACEI (%)	73	72	71	83	0.68
ARB (%)	13	19	9	8	0.426
Beta blocker (%)	82	84	76	92	0.419
Loop diuretic (%)	51	50	53	50	0.967
Thiazide diuretic (%)	1	0	3	0	0.519
Aldosterone antagonist (%)	28	38	24	17	0.283
Calcium channel antagonist (%)	5	3	3	17	0.144
Digoxin (%)	13	9	15	17	0.738
Ivabradine (%)	3	0	3	8	0.292
Creatinine (umol/L)	93 ± 24	96 ± 30	89 ± 19	95 ± 22	0.334
LVEF (%)	26.6 ± 7.0	27.2 ± 7.1	28.2 ± 6.4	23.5 ± 6.5	0.095
LVEDVi (mL/m2)	139 ± 35	137.2 ± 33.3	135.0 ± 40.5	152.0 ± 29.5	0.406
LVESVi (mL/m2)	104 ± 34	101.5 ± 33.8	97.5 ± 36.1	117.6 ± 29.2	0.133

#### Etiology of LVSD by combined cine plus LGE-CMR

Image quality was excellent or good in 98 and poor in 2 studies. Thirty-eight patients were classified as having ischemic, 46 non-ischemic and 16 dual pathology as the cause of LVSD (Table 2). Fifty-three patients had subendocardial or transmural hyperenhancement consistent with previous myocardial infarction. Forty-nine (80%) patients with NICM or dual pathology had evidence of mid-wall hyperenhancement on LGE-CMR; the other 13 had no visible LGE. The vast majority (*n* = 15, 94%) of patients with dual pathology had a combination of hyperenhancement patterns (subendocardial/transmural and mid-wall).

**Table 2:** Cause of LVSD diagnosed by LGE-CMR and perfusion-CMR

	LGE-CMR	PERFUSION-CMR	
Cause of LVSD (n)			
Ischemic	38	39	K = 0.968, *p* < 0.001
Non-ischemic	46	44	
Dual pathology	16	17	

#### Etiology of LVSD by complete stress perfusion-CMR studies

**Fig. 2:**
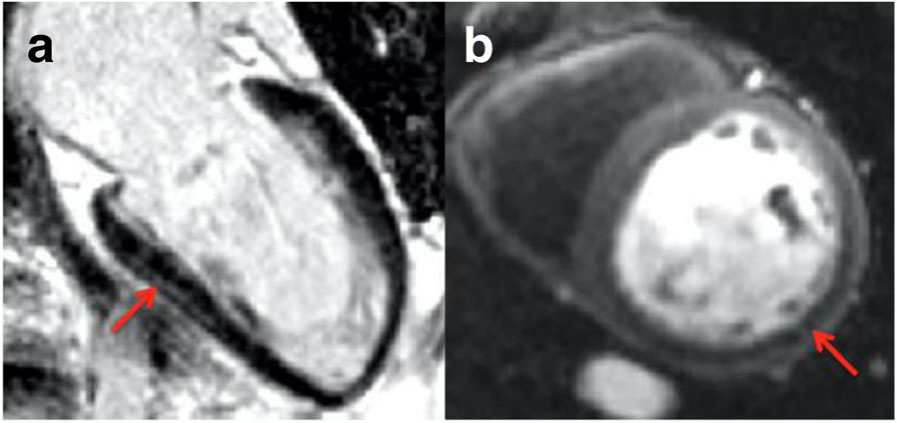
**a** Two-chamber LGE image with inferior LV mid-wall hyperenhancement (*arrow*). **b** First-pass perfusion-CMR image demonstrating an inferolateral subendocardial perfusion abnormality (*arrow*)

**Fig. 3:**
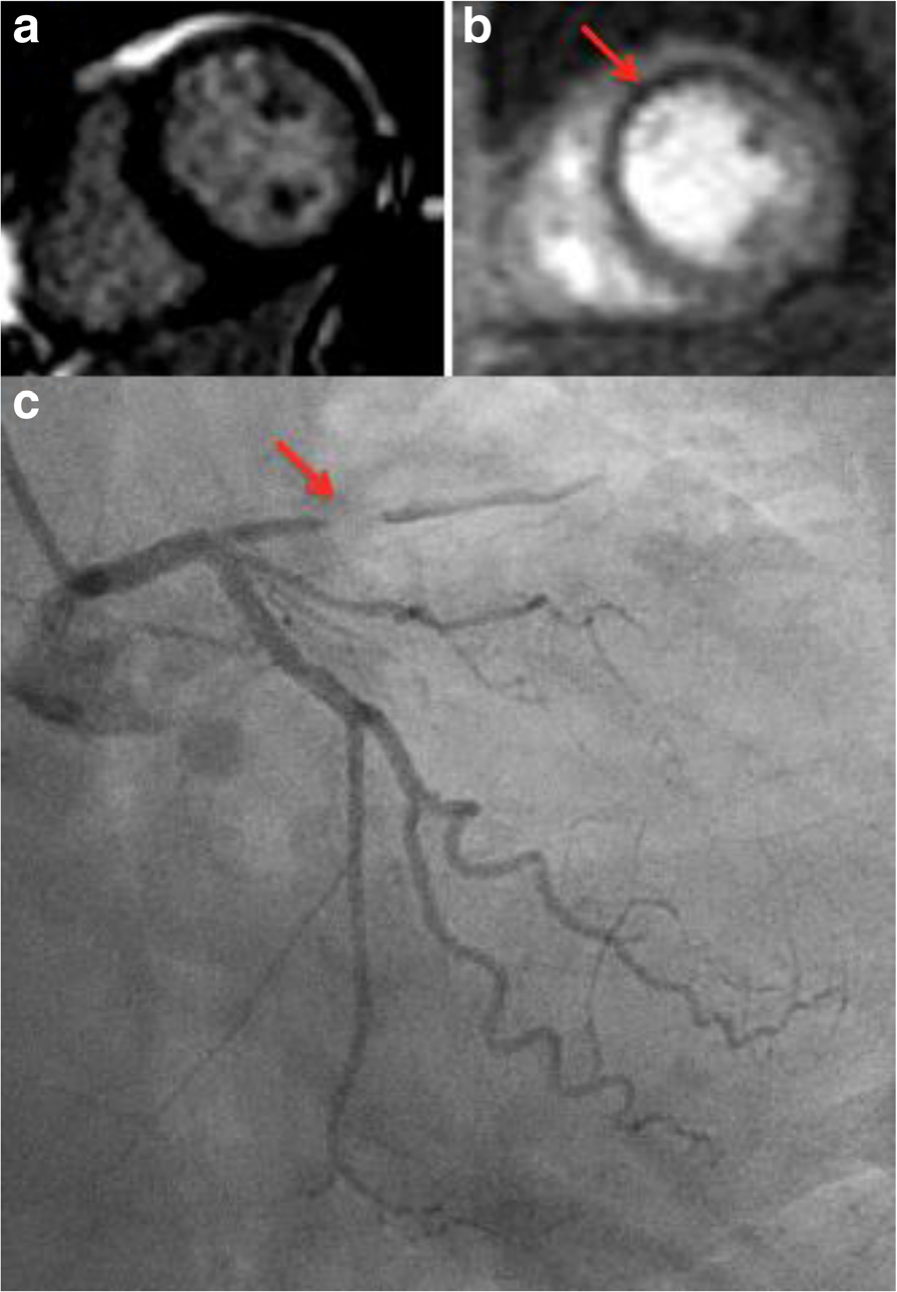
**a** Mid short-axis LGE window; there is no hyperenhancement to suggest myocardial infarction. **b** First-pass perfusion-CMR image showing a mid LV anteroseptal reversible perfusion defect (*arrow*). **c** The corresponding coronary angiogram image; there is a chronic total occlusion of the proximal-mid left anterior descending artery *(arrow*)

Image quality for first-pass perfusion was rated as good or excellent in 99 and poor in 1 patient. There were reversible perfusion defects in 23/54 (43%) patients with infarction and in 1 patient with no LGE. There was excellent agreement in diagnoses between LGE-CMR and perfusion-CMR (κ 0.968, *p*=<0.001) (Table 2), yielding a change in diagnosis from LGE-CMR in only 2 cases. In the first of these (78-year-old male, LV EF 26%), the LGE-CMR diagnosis was NICM. There was global LV hypokinesis with basal inferoseptal mid-wall LGE (Fig. 2a). The perfusion-CMR diagnosis was dual pathology as an inferolateral subendocardial perfusion abnormality was visualised, suggestive of ischemia (Fig. 2b), but not severe enough to account for the degree of LV impairment observed. In the second case (a 77-year-old female), the LGE-CMR diagnosis was NICM: image quality for LGE imaging was rated as poor (though still considered diagnostic), and no enhancement was visualised (Fig. 3a). The diagnosis by perfusion-CMR was ICM with demonstration of a subendocardial basal and mid LV anteroseptal perfusion defect (Fig. 3b). Subsequently coronary angiography revealed a significant LAD stenosis (Fig. 3c) and the patient underwent revascularisation.

#### Angiographic findings

Thirty-two of the patients underwent coronary angiography after CMR. The vast majority (*n* = 30, 93.8%) of these showed significant CAD. Both patients who underwent angiography that did not reveal significant CAD had LGE-CMR and perfusion-CMR diagnoses of NICM. LGE-CMR had a sensitivity of 87% for predicting significant CAD in subjects with severe LVSD, identifying ICM or dual pathology in 26/30 patients with significant CAD on angiography. Sensitivity of perfusion-CMR for predicting significant CAD was 90% (27/30 patients with significant CAD identified as having ICM or dual pathology). Only three cases of CAD identified by angiography were not detected by either LGE-CMR or perfusion-CMR (Table 3). In two of these, the degree of coronary disease was not deemed severe enough to alone account for the degree of LV impairment.

**Table 3:** Summary of angiographic and CMR findings in patients with angiographically-determined significant CAD and a diagnosis of NICM on CMR

Angiogram findings	Non-stress CMR findings	Perfusion-CMR findings	Comments
Moderate (50% stenosis) LCx disease.	Global LV hypokinesis. Severe bi-atrial dilatation with MR and TR. Mid-wall LGE present.	No perfusion abnormality detected.	CAD likely coincident and not main etiological factor.
Mild left mainstem (~30%) and severe RCA disease.	Global LVSD and marked intraventricular dyssynchrony. Severely dilated LA. Moderate MR. No LGE.	No perfusion abnormality detected.	CAD likely coincident and not main etiological factor. Patient condition improved with intensive medical therapy (now NYHA class I).
Three-vessel coronary disease. Severe LAD and LCx disease, moderate RCA disease.	Severe hypokinesis starting in the midanterior segment, becoming akinetic in the apex. No valve disease. No LGE.	No perfusion abnormality detected.	True false-negative CMR. Patient underwent CABG.

## Discussion

This study is the first to evaluate the incremental value of perfusion-CMR over LGE-CMR in identifying the etiology of heart failure in patients with severe LVSD. We have shown that the vast majority of patients with LVSD are identified by LGE and that the addition of stress perfusion only identified one additional patient with important ischemia requiring revascularisation.

### LGE-CMR versus perfusion-CMR

LGE-CMR is a proven technique for determining the etiology of heart failure [9], having been shown in previous studies to accurately differentiate between ICM and NICM [23, 24]. Clinical guidelines advocate the use of LGE-CMR in heart failure, when echocardiography is non-diagnostic [1, 25]. The utilisation of LGE-CMR in this context is known to have significant clinical impact; influencing patient management, clinical decisions and diagnoses in 65% of patients in one study [26]. When mid-wall LGE is present in NICM, this portends a poorer prognosis [13]. A high proportion of our patients with NICM had mid-wall LGE in comparison to the published literature (80 versus 30% in Gulati et al.) [13]. This is probably the result of our study group being older (69 (59–73) versus 51 ± 15 years) and with much poorer LV systolic function (mean LV EF 27.5 ± 6.8% versus 37.2 ± 13.1%) [13].

Perfusion-CMR is highly accurate for identifying myocardial ischemia due to CAD [15, 27, 28]. No published data exist, however, characterising the role of perfusion-CMR in determining the etiology of severe LVSD. Despite this, it is routine in our institution to undertake full adenosine stress perfusion-CMR studies for identifying the cause of heart failure in patients with newly diagnosed severe LVSD. The EuroCMR registry, which includes data on more than 27,000 consecutive CMR studies from over 15 European countries, showed that almost a third (29.3%) of CMR studies include adenosine stress perfusion imaging [16]. Data on the specific indications for perfusion-CMR are not presented in the EuroCMR registry, but it is likely that a significant number of these studies were also performed in subjects with severe LVSD.

Adenosine stress perfusion imaging adds time and expense to the CMR protocol. Seventy five to 100% of all complications associated with CMR occur due to administration of pharmacologic stress agents [16]. There is also evidence to suggest that patients with heart failure exhibit a diminished response to adenosine due to down regulation of adenosine receptors in the failing myocardium [16]. Clearly the role of perfusion-CMR in determining the etiology of heart failure should be subject to scrutiny before routine implementation in clinical practice.

We found excellent agreement between the causes of LVSD diagnosed by LGE-CMR and by perfusion-CMR, suggesting that perfusion-CMR is of limited additional benefit over LGE-CMR for the specific indication of identifying the etiology of heart failure. In only two of our 100 patients did perfusion-CMR alter the diagnosis established by LGE-CMR (and led to a meaningful change in patient management, i.e. revascularisation, in only one patient). Perfusion-CMR did, however, identify reversible ischemia in 43% of patients with a non-stress-CMR diagnosis of ICM/dual pathology. Whilst this did not alter the diagnosis in these patients it may have implications on clinical management. Generally both LGE-CMR and perfusion-CMR had excellent sensitivity for detection of CAD. Importantly these analyses were made entirely blinded to patient details and medical history, which in clinical practice would ordinarily guide risk stratification and decision-making and raise suspicion of CAD.

### Clinical implications

Our study demonstrates that stress perfusion-CMR is of minimal incremental benefit in diagnosing the cause of severe LVSD. It is therefore contentious whether stress testing should be routinely performed in this context. Patients with severe LVSD attributed to previous infarction on LGE-CMR with a likelihood of functional recovery often undergo coronary angiography and revascularisation. However the role of revascularization in patients with ICM in the absence of symptoms is controversial. In the STITCH trial, there was no significant difference in outcomes when patients with heart failure and coronary artery disease underwent surgical revascularization versus medical therapy alone [29]. In such instances perfusion-CMR is therefore of limited added benefit. In cases of severe LVSD where LGE-CMR excludes ICM by patterns of hyperenhancement specific to NICM, exposing the patient to risks of invasive angiography could be unwarranted as the likelihood of identifying significant coronary disease is low. On the other hand, we found that perfusion-CMR identified additional ischemia in half our patients diagnosed with ICM. In these cases, perfusion-CMR may influence subsequent management, although the benefit of routine revascularisation in the absence of angina over medical management is far from clear [29].

The present study challenges the incremental role of perfusion-CMR over LGE-CMR for diagnosing the etiology of heart failure in severe LVSD. Clinical guidelines do not specifically recommend perfusion-CMR for this purpose [1, 5]. However, observations from our own clinical practice and EuroCMR registry data suggest that perfusion-CMR is routinely utilised to determine the cause of severe LVSD when echocardiography is non-diagnostic [16]. Exposing patients in these cases to the added risks of adenosine infusion, together with increasing MR scanning times and expense are probably unjustified at present.

### Limitations

The retrospective and single-centre design limits the strength of this study, as does the relatively small sample size. Coronary angiography, as the reference standard for CAD, was not performed on all subjects in the cohort to exclude CAD. Computed tomography coronary angiography can be used to reliably exclude the presence of CAD but will be difficult to interpret in high risk patients with coronary calcium and does not provide prognostic information related to LGE [30].

## Conclusions

Adenosine stress perfusion-CMR is of minimal additional benefit to cine and LGE-CMR for determining the etiology of heart failure in patients with severe LVSD. Prospective studies are required to define the role of perfusion-CMR in heart failure and identify those patients most likely to benefit from the addition of perfusion imaging to LGE-CMR.

## Abbreviations

CAD: Coronary artery disease
CMR: Cardiac magnetic resonance
ICM: Ischemic cardiomyopathy
LGE: Late gadolinium enhancement
LVEF: Left ventricular ejection fraction
LVSD: Left ventricular systolic dysfunction
NICM: Non-ischemic cardiomyopathy
