# Correction to: does stress perfusion imaging improve the diagnostic accuracy of late gadolinium enhanced cardiac magnetic resonance for establishing the etiology of heart failure?

**DOI:** 10.1186/s12872-019-1001-y

**Published:** 2019-01-21

**Authors:** Gaurav S. Gulsin, Abhishek Shetye, Jeffrey Khoo, Daniel J. Swarbrick, Eylem Levelt, Florence Y. Lai, Iain B. Squire, Jayanth R. Arnold, Gerry P. McCann

**Affiliations:** 1 Department of Cardiovascular Sciences, University of Leicester, Glenfield Hospital, Groby Road, Leicester, LE3 9QP UK; 2 The NIHR Leicester Cardiovascular Biomedical Research Unit, Glenfield Hospital, Leicester, UK

**Correction to: BMC Cardiovascular Disorders (2017) 17:98.**

**DOI: 10.1186/s12872-017-0529-y**

Following publication of the original article [1], the author reported his name has erroneously spelled as Abishek Shetye. The correct name is Abhishek Shetye.

The publisher apologizes for any inconvenience caused by this error.
